# Role of Dietary Factors on DNA Methylation Levels of TNF-Alpha Gene and Proteome Profiles in Obese Men

**DOI:** 10.3390/nu16060877

**Published:** 2024-03-18

**Authors:** Chayanisa Boonrong, Sittiruk Roytrakul, Prapimporn Chattranukulchai Shantavasinkul, Piyamitr Sritara, Jintana Sirivarasai

**Affiliations:** 1 Doctoral Program in Nutrition, Faculty of Medicine Ramathibodi Hospital and Institute of Nutrition, Mahidol University, Bangkok 10400, Thailand; b.chayanisa@gmail.com; 2 National Center for Genetic Engineering and Biotechnology, National Science and Technology Development Agency, Pathum Thani 12120, Thailand; sittiruk@biotec.or.th; 3 Department of Medicine, Faculty of Medicine Ramathibodi Hospital, Mahidol University, Bangkok 10400, Thailand; sprapimporn@gmail.com (P.C.S.); piyamitr.sri@mahidol.ac.th (P.S.); 4 Nutrition Division, Faculty of Medicine Ramathibodi Hospital, Mahidol University, Bangkok 10400, Thailand

**Keywords:** TNF-α, DNA methylation, serum proteome, signaling pathway

## Abstract

Integrated omics-based platforms from epigenomics and proteomics technologies are used to identify several important mechanisms in obesity etiology, food components, dietary intake, regulation of biological pathways, and potential new intervention targets. Therefore, this study aimed to analyze whether dietary factors involved in the methylation of tumor necrosis factor (TNF)-α are implicated in differential protein expression in people with normal weight and obesity. Methods: The participants were classified into the non-obese (N = 100) and obese (N = 133) groups. DNA methylation levels of the TNF-alpha gene and proteomics were analyzed using the pyrosequencing method and LC-MS-MS, respectively. Results: Comparison between geometric means of DNA methylation of TNF-α showed lower levels in subjects with obesity than in those without obesity (*p* < 0.05). There were associations between dietary factors and some metabolic syndrome components and TNF-α DNA methylation levels. Proteomic analysis showed important signaling pathways related to obesity, with 95 significantly downregulated proteins and 181 upregulated proteins in the non-obese group compared with the obese group. Conclusion: This study shows an association between the dietary factors involved in the methylation of TNF-α and differential protein expression related to obesity. However, a large sample size in future studies is required to confirm our results.

## 1. Introduction

The incidence of obesity worldwide and its consequent economic burden are steadily increasing [1]. Therefore, there is an increasing need for the development of efficient therapeutic approaches. Currently, epigenetic modification is an important approach in precision medicine with a target for strategy development to personalized lines of treatment for obesity. Epigenetics is known as novel paradigm for exploring the mechanisms that contribute to the regulation of gene expression in diseases associated with obesity [2]. Abnormal increases or decreases in DNA methylation contribute to the development of metabolic diseases. DNA methylation of cytosines in cytosine-guanine dinucleotides (CpGs) is used as a biomarker to investigate epigenetic dysregulation of genes involved in inflammation, lipid and lipoprotein metabolism, and adipogenesis for obesity-induced hyper-or hypo-methylation and metabolic phenotypes [2]. An experimental study showed that a high fat intake induced overweight in rats along with increased adiponectin levels and changes in the methylation patterns in the promoters of key genes in fatty acid metabolism and liver steatosis [3].

Obesity is related to chronic low-grade inflammation, with the production of proinflammatory adipokines, such as tumor necrosis factor-α (TNF-α), interleukin-6 (IL-6), leptin, monocyte chemoattractant protein-1 (MCP-1), and resistin [4]. TNF-α is expressed in and secreted by adipose tissue, and its levels are associated with the degree of adiposity. TNF-α is causally linked to metabolic syndrome through various mechanisms. These mechanisms include the following: hyperglycemia via inhibition of insulin action; impairment of glucose clearance and hepatic glucose production; dyslipidemia via suppression of triacylglycerol clearance; promotion of insulin resistance-induced-hepatic de novo lipogenesis and stimulation of lipid synthesis and adipose lipolysis in the liver; and hypertension via alterations in renal hemodynamics, nephron transport, and the function of transporters [5,6,7]. A previous study of 165 subjects with overweight/obesity investigated the associations between inflammatory gene methylation, clinical blood parameters, and dietary data [8]. The results showed TNF-α methylation was negatively associated with cholesterol intake, but serum cholesterol concentrations were positively associated with TNF-α methylation [8]. However, an intervention study of an 8-week energy-restricted diet in individuals with obesity showed a strong association between the baseline TNF-α promoter methylation pattern and circulating TNF-α concentrations [9]. Changes in weight loss at the endpoint with total methylation of the TNF-α promoter suggested a putative role of epigenetic regulation of TNF-α expression in subjects with obesity and metabolic syndrome.

Integrated omics-based platforms from genomics, epigenomics, transcriptomics, proteomics, and metabolomics technologies have shown several main mechanisms in the etiology of obesity, regulation of biological pathways, and potential new intervention targets [10]. The serum or plasma proteome is widely used in clinical investigations because it is a reliable and efficient method of evaluating the molecular mechanisms underlying pathophysiological changes in obesity and driving personalized prevention [11]. A proteomic analysis of 1500 individuals with overweight/obesity showed a group of proteins associated with body mass index (BMI) and closely associated with chronic inflammation, such as complement factor, C-reactive protein (CRP), and proline-rich acidic protein 1 [12]. Furthermore, an epigenome-wide association study of a population-based cohort of 3080 participants with proteomic data showed associations of 14 of the proteins and 8 of the CpG sites with relevant clinical phenotypes, including BMI, alcohol consumption, total cholesterol, high-density lipoprotein (HDL), low-density lipoprotein (LDL), and triglycerides (TGs) [13].

Nutrigenomics is currently used extensively in novel investigations involving obesity and dietary-related diseases because it addresses the effect of nutrients or nutritional supplements on gene and protein expression [14]. There is a link between DNA methylation of TNF alpha and proteomic in plasma. Changes in DNA methylation of TNF alpha may be both causal for changes in biological processes by differentially regulating gene expression and consequential in response to modified physiological or environmental factors including diet and disease, such as obesity or low-grade inflammation. Blood circulating proteins, as intermediate phenotypes, can reveal the underlying disease-causing pathways in obesity [14]. We hypothesize that dietary factors modify the DNA methylation of inflammatory genes in obese individuals, and these molecular changes contribute to the more complex phenomenon of proteomic profiles. Therefore, this study has the goal of evaluating the relationship between dietary factors implicated in TNF-α methylation and differential protein expression in normal-weight and obese individuals.

## 2. Materials and Methods

### 2.1. Study Participants

Participants (men aged 45–60 years) in this study were individuals who participated in the cohort study called the Electricity Generating Authority of Thailand (EGAT) in collaboration with Mahidol University, Thailand. We collected data from a health survey (EGAT2/5) in 2018 that were related to this study. These data included age, educational level, occupation, tobacco smoking, alcohol drinking, medical history and family history of cancer, cardiovascular disease, other metabolic diseases (diabetes, hypertension, and dyslipidemia), and inflammatory disease, which were recorded on the same day through a health examination, measurement of anthropometry, a dietary assessment, and biochemical analysis. The exclusion criteria were individuals with hypertension, diabetes, chronic kidney disease, chronic liver disease, chronic inflammatory disease, cardiovascular disease, cancer, thyroid disease, or gastrointestinal disease. Metabolic syndrome was diagnosed in accordance with the National Cholesterol Education Program Adult Treatment Panel III definition if three or more of the following five criteria were met: abdominal obesity (waist circumference ≥90 cm), high blood pressure (BP) (systolic/diastolic BP ≥130 or ≥85 mmHg, respectively), hyperglycemia (fasting blood glucose concentrations ≥100 mg/dL), hypertriglyceridemia (TG concentrations ≥150 mg/dL), and low (HDL)-cholesterol (HDL-C) concentrations (<40 mg/dL) [15]. The study was conducted according to the guidelines of the Declaration of Helsinki, and approved by the Ethics Committee of Faculty of Medicine Ramathibodi hospital, Mahidol University (protocol code COA. No. MURA2019/67 and date of approval; 11 February 2019).

### 2.2. Anthropometric Measures

Trained staff performed anthropometric measurement of weight and height. BMI values were calculated. Height (cm) and body weight (kg) were measured in light clothes without shoes. In this study, the participants in the normal weight (non-obese) and obese groups were classified according to the World Health Organization recommendations for Asian populations which state a BMI of 18.5–22.9 kg/m^2^ for normal weight (non-obese) and ≥ 25 kg/m^2^ for obesity [16]. The participants’ BMI was estimated as weight in kg divided by the square of height in meters (kg/m^2^). Waist circumference was measured unclothed using a tape measure and was measured in the horizontal plane of the superior border of the iliac crest.

### 2.3. Assessment of Dietary Intake

A self-reported semi-quantitative food frequency questionnaire (FFQ) was used for collecting dietary data. The FFQ consisted six food groups. There were 24 questions related to protein, 3 to fruits, 8 to vegetables, 12 to carbohydrates, 7 to dairy products, 12 to drinks and beverages, 5 to fats (for cooking), and 6 to miscellaneous items. After trained staff explained the portion size of each product to the participants, they were asked to recall their average frequency of dietary consumption of the standard serving size during the previous 12 months. The study participants indicated the frequency of all consumed beverages and food items on a scale ranging from 0 to 3 times/week, 4 to 6 times/week, and >6 times/week. The frequency per week for egg, fruits, and vegetable intake was reported as 0–3 servings, 4–6 servings, and >6 servings.

### 2.4. Biochemical Analysis

Blood samples were obtained from all participants after an overnight fast at the same time as the heath examination. These blood samples were promptly processed to perform DNA methylation and biochemical analyses. Biochemical parameters, such as the lipid profile (consisting of TGs, total cholesterol, HDL-C, LDL-C), fasting plasma glucose (FPG), glycated hemoglobin, alanine transminase, aspartate transaminase (AST), blood urea nitrogen, and creatinine, were analyzed by an automatic analyzer (Cobas-Mira; Roche, Milan, Italy)

### 2.5. DNA Methylation Analysis

A volume of 3 mL of whole blood was collected into EDTA tubes from each participant and centrifuged at 2500 g for 15 min. The buffy coat fraction was transferred to a cryovial and immediately frozen at −80 °C until use. A DNA extraction and purification kit (Promega, Madison, WI, USA) was used, following the manufacturer’s instructions. DNA (500 ng) underwent bisulphite conversion, with an efficiency of >99%, using an EZ DNA methylation kit (ZymoResearch, Inc., Irvine, CA, USA) to convert un-methylated cytosine bases (C) into uracil bases (U), according to the manufacturer’s protocol. M-Elution Buffer (ZymoResearch, Inc., Irvine, CA, USA) was used to elute bisulfite-treated DNA. Polymerase chain reaction (PCR) pyrosequencing-based assays were used for measuring %5-methylcytosine at the four CpG position in the gene promoter region at chromosome 6 (position 1: 2874672, position 2: 2874678, position 3: 2874680, and position 4: 2874695), as previously described [8]. Pyrosequencing assay information for TNF-α consisted of the following: forward primer, biotin-TGAGGGGTATTTTTGATGTTTGT; reverse primer, TTAATAATTATTTTTATATATTTT; and sequencing primer, ATAAATTTTA TATTTTTTAT. PCR cycling conditions were 95 °C for 60 s, 56 °C for 60 s, and 72 °C for 60 s (50 cycles).

In brief, a 50 μL PCR reaction was carried out with 25 μL of GoTaq Green Master Mix (Promega), 1 pmol of forward primer, 1 pmol of biotinylated reverse primer, and 500 ng of bisulfite-treated genomic DNA. The PCR product was bound to streptavidin high-performance sepharose (Amersham Biosciences, Uppsala, Sweden). The sepharose beads containing the immobilized PCR product were purified, washed, and denatured using 0.2 M NaOH solution and rewashed using the Pyrosequencing Vacuum Prep Tool (Pyrosequencing, Inc., Westborough, MA, USA), as recommended by the manufacturer. Pyrosequencing primer (0.3 μM) was annealed to the purified single-stranded PCR product, and pyrosequencing was performed with the PyroMark MD System (Pyrosequencing, Inc.). The degree of methylation was expressed as the percentage of cytosines that were methylated. This percentage was calculated as the number of methylated cytosines divided by the sum of methylated and unmethylated cytosines, multiplied by 100% (%5-methylcytosine). Each sample was measured three times to increase the reliability and to increase the precision of the findings.

### 2.6. Protein Quantitation and Identification by Liquid Chromatography–Mass Spectrometry (LC-MS/MS)

A proteomic analysis was performed using LC-MS/MS, as described previously [17]. A total of 20 µg of pooled serum protein was obtained from 100 and 133 subjects in the non-obese and obese groups, respectively. Total protein was extracted and the protein content was evaluated by the Lowry method, and in-gel digestion was performed as described previously [18]. MaxQuant 1.6.1.12 was used to quantify the proteins in individual samples using the Andromeda search engine to correlate MS/MS spectra with the UniProt Homo sapiens database. The following parameters were used for data processing: a maximum of two miss cleavages, mass tolerance of 20  ppm for the main search, trypsin as the digesting enzyme, carbamidomethylation of cysteines as fixed modification, and the oxidation of methionine and acetylation of the protein N-terminus as variable modifications. Only peptides with a minimum of seven amino acids as well as at least one unique peptide were required for protein identification. Only proteins with at least two peptides and at least one unique peptide were considered to be identified and used for further data analysis. ShinyGO 0.77 software (http://bioinformatics.sdstate.edu/go, accessed on 20 April 2023) was used for in-depth analysis with graphical visualization of enrichment, and we also used the Kyoto Encyclopedia of Genes and Genomes (KEGG) pathway related to the identified proteome. In addition, MetaboAnalyst (https://www.metaboanalyst.ca, accessed on 20 April 2023), which is a web-based platform dedicated to comprehensive omics data analysis and interpretation, was applied for comparison of serum proteome profiles between the non-obese and obese groups. The results are presented as a volcano plot and up/downregulated proteins.

### 2.7. Statistical Analysis

Statistical analyses were performed using IBM SPSS version 23 (IBM Corp., Armonk, NY, USA). Continuous and categorical variables are presented as the mean ± standard deviation and frequency (%), respectively. The normality of each variable was evaluated using the Shapiro–Wilk test. Methylation levels of TNF-α were log-transformed and expressed as geometric means and the standard deviation. Between-group comparisons for anthropometry, metabolic profiles, and other variables were conducted using an unpaired Student’s *t*-test. Univariate and multivariate (adjusted for metabolic syndrome components) regression analyses with categorical predictors were performed using the general linear model. Further analysis was performed to compare the estimated marginal means of the total DNA methylation between the non-obese and obese groups. Estimated marginal means provided estimates of predicted mean values (total DNA methylation level) in the model between the non-obese and obese groups with and without metabolic syndrome. Interaction plots of these means were created to visualize some of the relationships. All statistical tests were two-sided, and a *p*-value < 0.05 was considered statistically significant.

## 3. Results

### 3.1. Characteristics of the Participants

In this study, 233 participants were classified into the non-obese (normal BMI) (N = 100) and obese (N = 133) groups. The participants had a mean BMI of 21.47 ± 1.03 kg/m^2^ and 28.46 ± 3.27 kg/m^2^, respectively. Descriptive characteristics and biochemical profiles of the participants are shown in Table 1. Subjects in the obese group had a significantly higher WC, SBP, and DBP than those in the non-obese group (all *p* < 0.05). Current smoking and drinking statuses were not different between the groups. In addition, FPG, glycated hemoglobin, TG, LDL-C, and AST concentrations were significantly higher in the obese group than in the non-obese group (*p* < 0.05). However, HDL-C concentrations were lower in the obese group than in the non-obese group.

### 3.2. Associations between DNA Methylation of TNF-α and Metabolic Components and Dietary Factors

The geometric mean values of the four positions and total levels of DNA methylation of TNF-α were significantly lower in the obese group than in the non-obese group, as shown in Table 2 (all *p*-values < 0.05). In addition, we performed a further analysis to determine the differences in each position and total DNA methylation of TNF-α levels in relation to metabolic components in the non-obese and obese groups (Table 2). The non-obese and obese groups with TG concentrations ≥150 mg/dL showed some significantly lower positions and total levels of DNA methylation of TNF-α than those with TG concentrations < 150 mg/dL. We found significant differences in DNA methylation of TNF-α at position 4 (19.46 ± 4.43% vs. 21.63 ± 4.36%) and total levels (55.34 ± 11.67% vs. 58.44 ± 10.01%) in the obese group between subjects with and without hypertension. A similar trend was also found in the obese group with FPG concentrations ≥110 mg/dL compared with those with FPG concentrations <10 mg/dL for DNA methylation of TNF-α at position 2 (12.21 ± 2.89% vs. 14.97 ± 2.91%), position 4 (21.79 ± 4.24% vs. 24.47 ± 4.45%), and total levels (58.25 ± 10.02% vs. 61.36 ± 10.62%) (all *p* < 0.05).

We also evaluated the association between dietary factors and DNA methylation of TNF-α levels in the two study groups (Table 3). The regression coefficient of dietary variables from univariate and multivariate analyses for predicting log DNA methylation of TNF-α levels showed some significant associations. Red meat, processed meat, and fried meat intake were negatively associated, whereas egg, fruit, and vegetable intake were significantly positively associated with log DNA methylation of TNF-α levels in the non-obese and obese groups.

**Figure 1:**
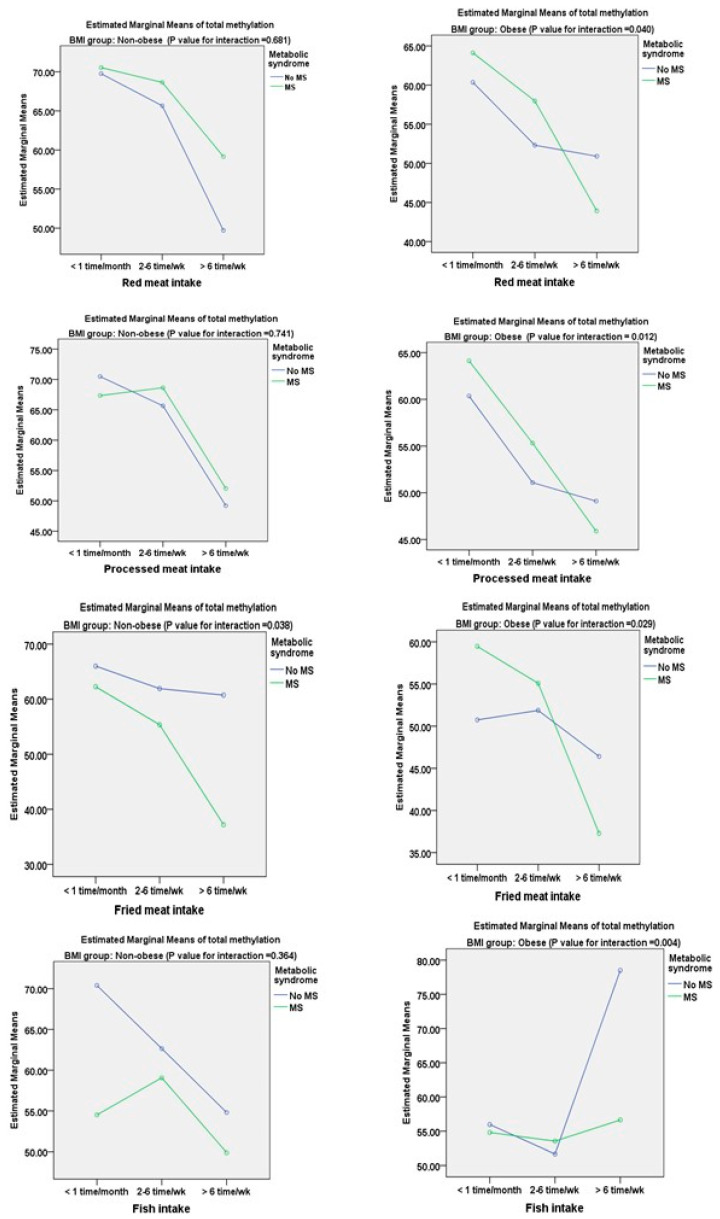
Estimated marginal means of total methylation classified by red meat, processed meat, fried meat, and fish intake among non-obese and obese groups.

**Figure 2:**
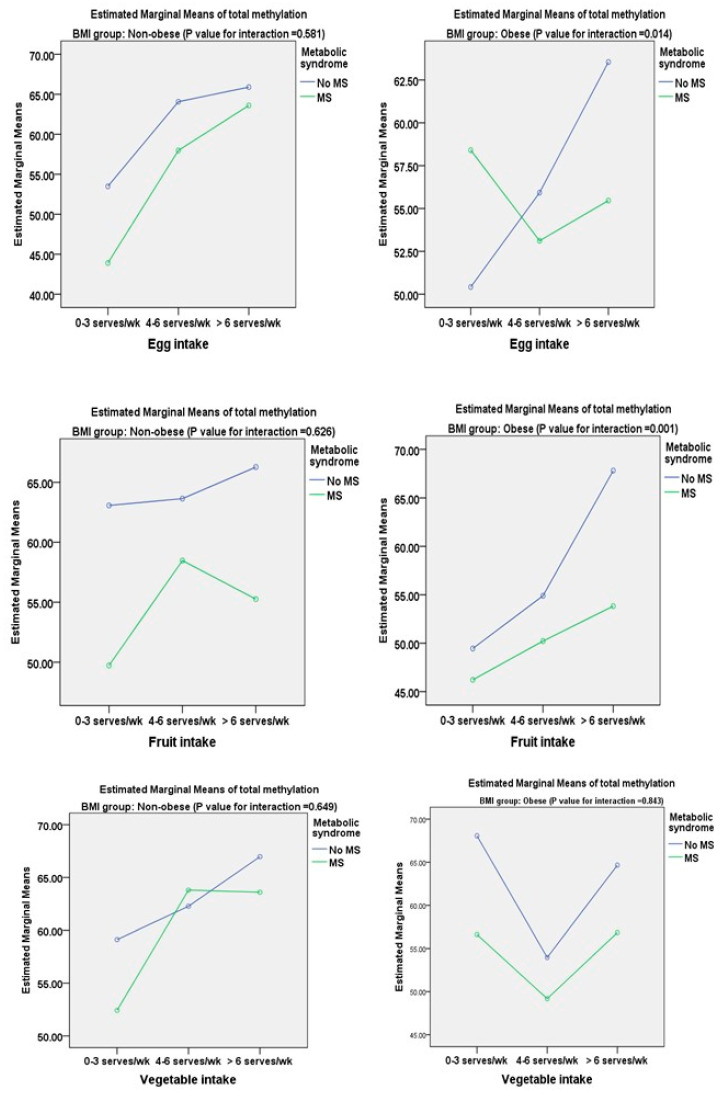
Estimated marginal means of total methylation classified by egg, fruit, and vegetable intake among non-obese and obese groups.

**Figure 3:**
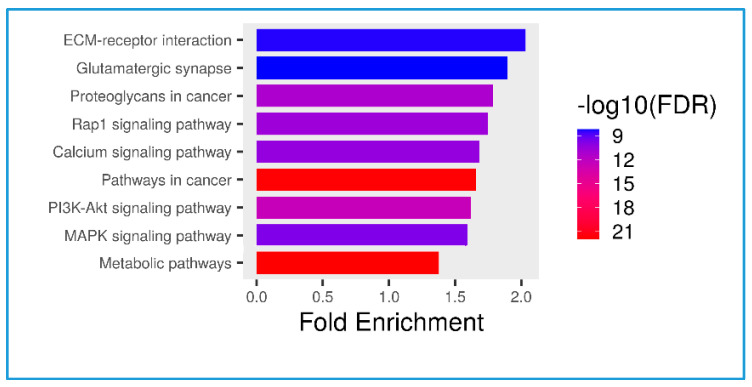
Pathway analysis indicating the top ten KEGG pathways related to serum total proteomes in this study.

The estimated marginal means of total methylation from the general linear model of dietary intake of the participants are shown in Figure 1 and Figure 2. In this study, the numbers of non-obese individuals without MS and with MS were 84 and 16, respectively, whereas there were 35 cases of metabolically healthy obesity (26.3%) and 98 case of obesity with MS (73.68%). There was a significant decrease in the estimated marginal mean of total DNA methylation with an interaction between the consumption frequency and participants with and without metabolic syndrome only for fried meat in the non-obese group (*p* = 0.038). However, in the obese group, this decrease in total DNA methylation was also found for red meat (*p* = 0.040), processed meat (*p* = 0.012), and fried meat (*p* = 0.029). There was a significant increase in total DNA methylation with the consumption frequency of fish intake in the obese group (*p* = 0.004). The estimated marginal mean of total DNA methylation was significantly increased with a greater frequency of egg (*p* = 0.014) and fruit (*p* = 0.001) intake in the subjects in the obese group without metabolic syndrome compared with those with metabolic syndrome (Figure 3). In the non-obese group, there were no significant interactions between egg, fruit, and vegetable intake and the metabolic syndrome status.

### 3.3. Serum Proteomic Analysis, Differential Protein Expression, and Potential Mechanisms Related to Identified Proteins in the Non-Obese and Obese Groups

**Figure 4:**
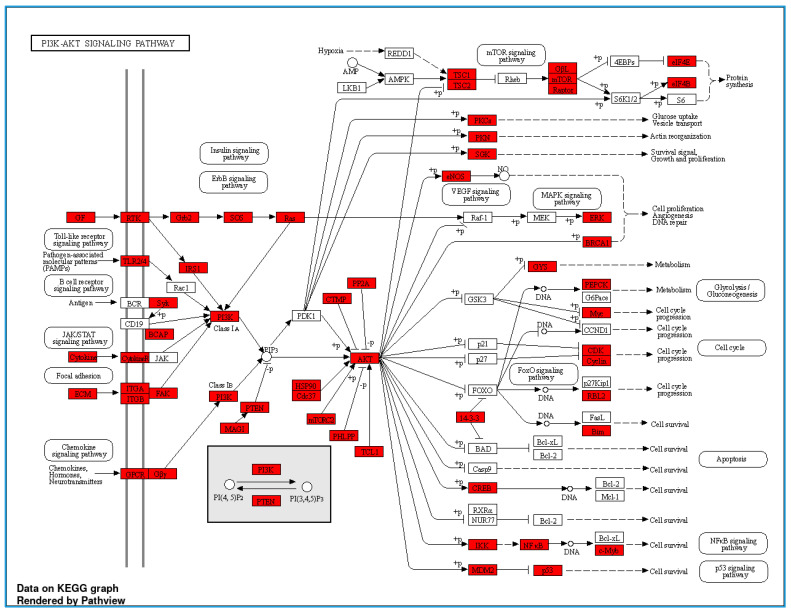
Serum proteomes of the study population related to the PI3K-Akt signaling pathway (the red block represents some proteins identified in the serum of the study population).

In the proteomic analysis, we identified the serum proteome in the non-obese and obese groups. Figure 3 shows a pathway analysis (N = 12) of the total serum proteins in this study. The KEGG pathways with fold enrichment from high (2.03) to low (1.07) values were extracellular matrix–receptor interaction, glutamatergic synapse, proteoglycans in cancer, the Rap1 signaling pathway, the calcium signaling pathway, pathways in cancer, the phosphatidylinositol 3-kinase (PI3K)-Akt signaling pathway, the mitogen-activated protein kinase (MAPK) signaling pathway, and metabolic pathways. We further analyzed the PI3K-Akt signaling pathway because of its close relevance to inflammation in obesity (Figure 4).

**Figure 5:**
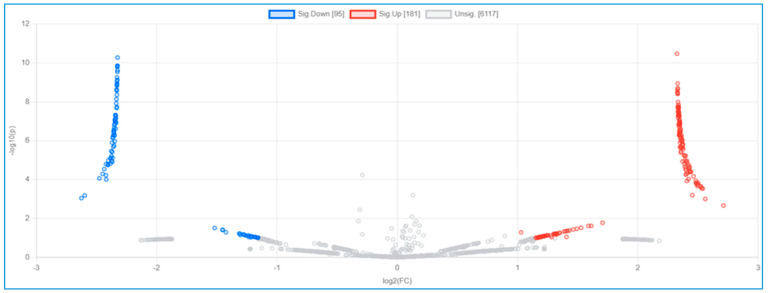
The 95 downregulated proteins and 181 upregulated proteins between the non-obese group and obese group.

We compared the serum proteome profiles between the non-obese and obese groups. A volcano plot shows 95 significantly downregulated proteins and 181 upregulated proteins in the non-obese group compared with the obese group (Figure 5). The top 10 downregulated proteins were intercellular adhesion molecule 4, phosphatase and actin regulator, histone-lysine N-methyltransferase 2C, gamma-tubulin complex component 6, caspase recruitment domain-containing protein 11, RNA-binding motif protein 42, putative zinc finger protein 487, tubulin tyrosine ligase like 8, cyclin-dependent kinase 15, and protein FAM227B. The top 10 upregulated proteins were coiled-coil and C2 domain-containing protein 2A, autophagy and beclin 1 regulator 1, voltage-dependent P/Q-type calcium channel subunit alpha, glutamate-rich protein 6, tuftelin-interacting protein 11, KIAA0232, spectrin repeat-containing nuclear envelope family member 3, major histocompatibility complex (MHC) class II antigen, nicotinamide nucleotide adenylyltransferase 3, and mediator of RNA polymerase II transcription subunit 13. These proteins’ log 2-fold changes and *p*-values are shown in Table 4. A comparison of 276 significantly up- and downregulated proteins between the non-obese and obese groups is shown in Supplementary Table S1. Based on data of identified proteins in Table 4, some proteins have been reported in the PI3K-Akt signaling pathway/the inflammatory process associated with the TNF-alpha function (https://www.uniprot.org/, accessed on 10 April 2023; http://stitch.embl.de/, accessed on 10 April 2023; and https://genemania.org/, accessed on 20 April 2023). In terms of proteins associated with PI3K-Akt signaling pathway, there were T2B5D4 (CMyc splice variant), M0R275 (AKT serine/threonine kinase 2), A0A075B6T1 (Autophagy and beclin 1 regulator 1), O95597 (Bcl−2-JH protein), H7C413 (BRCA1/BRCA2-containing complex subunit 3), F8WDP7 (cyclin-dependent kinase 15), A0A0S2Z592CDK5 regulatory subunit-associated protein 1 isoform 1, B1AVT0 (CDC-like kinase 2), A0A2K8FKR1 (phosphatidylinositol-4,5-bisphosphate3-kinase), A8KA75 (phosphatidylinositol-4,5-bisphosphate 3-kinase), Q96BE9 (cyclin-dependent kinase 4), Q5MAI5 (cyclin-dependent kinase-like 4), and Q53FZ9 (Fas-activated serine/threonine kinase isoform 1 variant). The other group of identified proteins related to the inflammatory process, and TNF-alpha pathway included M0R0X1 (RAB4B, member RAS oncogene family), E7EMV7 (TNFAIP3 interacting protein 1), Q12986 (transcriptional repressor NF-X1) and F8W8G5 (MYB proto-oncogene, transcription factor).

## 4. Discussion

The chronic inflammatory state associated with obesity can be responsible for some deleterious health consequences. DNA methylation plays a critical role in the regulation of gene expression through recruiting proteins involved in gene repression or by inhibiting the binding of transcription factor(s) to DNA [19]. In addition, an increased secretion of cytokines such as TNF-α has been reported with obesity-associated adipose tissue enlargement [2]. In this study, we found significantly lower levels of TNF-α DNA methylation (for four positions and total levels) in the obese group than in the non-obese group. A previous study on women with high truncal fat showed significantly lower 5-methylcytosine levels (*p* < 0.05) of the TNF-α gene promoter than those in women with lower truncal adiposity [20]. In addition, higher circulating TNF-α levels were correlated with methylation levels in the women with greater truncal adiposity These findings suggest that molecular mechanisms are involved in body fat accumulation and subsequent low-grade inflammation over time.

In recent years, various aspects of metabolic syndrome and its associated epigenetic changes have been investigated. Metabolic features (e.g., high BMI or low-grade inflammation or low antioxidant status) and dietary factors related to gene expression regulation in obesity-related diseases have been studied [10,21]. In this study, we found differences in DNA methylation related to each metabolic syndrome component in the non-obese and obese groups. We also found significantly lower DNA methylation levels of TNF-α in subjects with obesity and a WC ≥ 90 cm, TG concentrations ≥ 150 mg/dL, high blood pressure (systolic BP ≥ 130 mmHg or diastolic BP ≥ 85 mmHg), and FPG concentrations ≥ 110 mg/dL compared with those without MS (Table 2). Our findings were similar to those in a previous report that outlined the associations between TNF-α promoter methylation levels and several anthropometric variables (BMI, WC, and total body fat) and different metabolic features (e.g., total cholesterol–HDL-c and LDL-C–HDL-C ratios), which suggested a TNF-α regulatory action on obesity related to metabolic syndrome [8]. However, another study showed no difference in high-sensitivity CRP or TNF-α levels between metabolically healthy individuals who were morbidly obese and metabolically unhealthy individuals who were morbidly obese [22]. An animal study showed that increased plasma TNF-α concentrations possibly activated NADPH oxidase, resulting in the release of high superoxide levels from polymorphonuclear leukocytes and the possible development of hypertension through increased systemic oxidative stress and vascular tone [23]. Abnormal FPG concentrations and the risk of insulin resistance related to TNF-α may involve the generation of reactive oxygen species and impairment of insulin signaling through serine phosphorylation, which leads to the development of type 2 diabetes mellitus [24]. Furthermore, TNF-α promotes lipolysis and reduces insulin sensitivity by activating nuclear factor kappa B and c-Jun N-terminal kinase [25].

We also evaluated the association between dietary intake derived from the semi-FFQ and DNA methylation of TNF-α in the non-obese and obese groups. Univariate and multivariate analyses showed significant positive associations between TNF-α methylation and dietary intake of fish, egg, fruits, and vegetables. We also found negative associations between TNF-α methylation and dietary intake of red meat, processed meat, and fried meat (Table 3). We further compared estimated marginal means of TNF-α DNA methylation with frequency of food consumption in subjects with and without metabolic syndrome. Significant findings related to all subtypes of food intake, except for vegetable intake, were mostly found in the obese group (Figure 1 and Figure 2). A previous study showed that TNF-α DNA methylation was associated with the intake of several foods or nutrients, such as cholesterol, folic acid, beta-carotene, carotenoid, and retinol [8]. A cross-sectional study investigated the associations between processed and unprocessed red meat consumption and plasma inflammation markers [26]. This previous study showed that processed meat consumption was significantly associated with higher IL-6 concentrations, but not with CRP concentrations, and it was inversely associated with total TNF-α concentrations A meta-analysis showed that egg consumption (the quantity of eggs consumed in the intervention was 1–2 eggs/day and >2 eggs/day) had no significant effect on serum biomarkers of inflammation (high-sensitivity CRP, IL-6, and TNF-α) in adults [27]. In contrast to our finding, Ballesteros et al. [28] found a decrease in plasma concentrations of AST and TNF-α following egg intake (one egg/day for week). This finding might be explained by the presence of lutein and zeaxanthin in egg yolk. The effects of increased fruit and vegetable consumption on a significant decrease in high-sensitivity CRP and TNF-α concentrations were reported in a community-based study (N = 965) [29]. Furthermore, in healthy young adults, the highest tertile of energy-adjusted fruit and vegetable consumption (>660 g/d) was associated with lower plasma CRP concentrations and lower TNF-α gene expression from peripheral blood mononuclear cells [30]. In another study, TNF-α and IL-6 concentrations and myeloperoxidase activity were higher (whereas catalase and superoxide dismutase activities were lower) in consumers with a high intake of ultra-processed food (UPF) with metabolic syndrome than in those with a low intake of UPF [31]. These findings indicate an association between dietary consumption and the proinflammatory status and suggest a new aspect involved in the mechanisms related to nutrigenomics. An important issue should be considered in our findings: DNA methylation changes have a significant impact on dietary factors. In fact, various nutrients play a role as methyl donors or participate in DNA methylation maintenance, or directly affect the enzymes involved in the methylation process [32].

A multifaceted strategy is required for preventing or controlling metabolic disorders because of the complex interplay of environmental, genetic, and epigenetic factors in the development of metabolic diseases [33]. We investigated the differential expression of serum proteome profiles and possible mechanisms in the non-obese and obese groups (Figure 4 and Figure 5 and Table 4). On the basis of the top 10 KEGG pathways identified from total serum proteins, we focused on the PI3K-Akt pathway, owing to a large number of identified proteins (Figure 5) coded by genes in this signaling pathway. Cytokines bind to a specific receptor on the surface of their target cell and are constitutively associated with protein members of the JAK/STAT signaling pathway, subsequently transactivating the PI3K-Akt signaling pathway [34]. Our results on serum proteins provide evidence for the role of the PI3K protein family being responsible for abnormal metabolic disease downstream of PI3K in the glucose, insulin, and lipid signaling pathways [35]. Upregulation and downregulation of PI3K/AKT can be beneficial in obesity depending on the context. We further compared upregulated and downregulated proteins between the non-obese and obese groups (Figure 5 and Table 4). Some proteins have previously been reported to have important roles related to obesity, similar to the results of our study.

In this study, intercellular adhesion molecule 4 was upregulated in the obese group. This protein plays a role in blood coagulation and thrombosis. Ihanus et al. [36] found that intercellular adhesion molecule-4/integrin interactions related to erythrocytes activated neutrophils and monocytes, which resulted in the development of thrombus. In addition, a study on changes in adipose tissue gene expression in a randomized trial featuring diet, exercise, or a combined diet plus exercise regime in controls and postmenopausal women with overweight/obesity showed that decreased intercellular adhesion molecule-4 expression alongside weight loss was associated with Jak-STAT signaling [37]. CARD11 is a membrane-associated guanylate kinase superfamily, which mediates nuclear factor-κB activation through functions upstream of the IκB-kinase complex and cooperates with Bcl10 in a CARD domain-dependent manner [38]. The cross-talk between adipocytes and adipose tissue macrophages initiates cell death machinery and nuclear factor-κB-mediated inflammation, eventually resulting in ectopic lipid deposition, glucose intolerance, and other metabolic complications [39]. Another protein in our results was CDK15, a cyclin-dependent kinase which is involved in the integration of extracellular and intracellular signals for modulating gene transcription and cell division. A previous study on an animal model with analysis of CDK gene expression during adipocyte differentiation using a quantitative polymerase chain reaction found that CDK15 increased with adipocyte differentiation, reaching a peak on the fourth day and then decreasing [40].

For the downregulated proteins found in this study, AMBRA1 is involved in the PI3K complex I that controls autophagosome formation and plays an important role in mitophagy, which is the efficient turnover of mitochondria [41]. AMBRA1 has been reported to have various functions in carcinogenesis, including autophagy, tumorigenesis, proliferation, and the cell cycle. Emerging evidence has indicated that AMBRA1 affects cancer formation, maintenance, and progression by regulating c-MYC and cyclins, which are frequently deregulated in human cancer cells [42]. CACNA1A is a protein in voltage-dependent Ca^2+^ channels that controls the entry of Ca^2+^ into excitable cells and regulates BP through vascular smooth muscle contraction [43]. A genome-wide association meta-analysis of systolic BP, diastolic BP, and hypertension (N = 86,588 individuals) showed that genetic polymorphisms of CACNA1A were potentially associated with changes in BP and hypertension [44]. MHC class II antigen was another protein found in our study. A previous study showed that the MHC class II pathway mediated adipose inflammation, as shown by a microarray analysis of primary adipocytes [45]. Additionally, multiple genes involved in MHCII antigen processing and presentation were increased in obese women.

This study showed associations between dietary factors involved in the methylation of TNF-α and differential protein expression in the non-obese and obese groups, and these were related to metabolic syndrome. Promoter methylation patterns are potential biomarkers that could be applied for managing obesity, and TNF-α gene expression in blood could be partly determined by dietary factors. At present, knowledge about DNA methylation and inflammation is not sufficiently detailed (and their exact mechanisms are unknown), but it could be partially supported by our proteomic analysis. The overall findings from this study emphasize the role of nutrigenomics, with two central platforms—epigenetics and proteomics—addressing the interactions between nutrients, bioactive compounds in foods, and genes (and how these interactions influence phenotype, including risk of developing obesity).

However, there are some limitations to this study. Peripheral blood is one of the most common biological matrices for epigenetic investigations, but DNA methylation levels measured in blood cells result from a mixture of different cell types. It is difficult to know whether the differences obtained between obese and non-obese groups are due to differences in cell composition in both groups or due to altered DNA methylation patterns in the TNF-α promoter. Future epigenomics studies should be performed with different cellular composition analyses. In addition, we did not measure TNF-α concentrations, and this limited our evidence supporting the association between an epigenetic and clinical or biochemical phenotypes related to obesity. This cross-sectional study with a small sample size could not define a cause-and-effect relationship. Large prospective cohort studies are required to confirm our results. Finally, dietary assessment using the semi-FFQ is the most common method used in nutrition research, but it is not precise enough to measure absolute intake of different food components. Further study with a combination of methods, such as the FFQ with a 24 h dietary record or the FFQ with biomarker levels, may be used to obtain more accurate estimates of dietary intake than those of individual methods.

## 5. Conclusions

In this study, DNA methylation of TNF-α profiles was significant associated with obesity, metabolic syndrome components (abnormal waist circumference, TG, and FPG), and dietary factors. These findings demonstrate the occurrence of epigenetic mechanisms involving the regulation of TNF-α expression. In addition, the proposed signaling pathways that form our proteomic analysis include PI3K and inflammatory response related to TNF-α, which contribute to am interrelationship between DNA methylation of inflammatory genes, differential protein expressions, and implications for obesity-induced metabolic disorders.

## Figures and Tables

**Table 1:** General characteristics and biochemical parameters of the study population.

Characteristics	Total (N = 233)	Non-Obese Group (N = 100)	Obese Group (N = 133)
Age (years)	57.70 ± 1.41	57.68 ± 1.41	57.71 ± 1.42
BMI (kg/m^2^)	25.46 ± 4.30	21.47 ± 1.03	28.46 ± 3.27 ^a^
Waist circumference (cm.)	92.9 ± 10.87	83.38 ± 5.01	100.07 ± 8.29 ^a^
SBP (mmHg)	138.04 ± 16.90	133.70 ± 16.20	141.30 ± 16.72 ^a^
DBP (mmHg)	79.00 ± 9.45	76.21 ± 9.02	81.10 ± 9.25 ^a^
Smoking, n (%)			
Non-smoker	109 (46.8%)	52 (52.0%)	57 (42.9%)
Smoker	31 (13.3%)	16 (16.0%)	15 (11.3%)
Ex-smoker	93 (39.9%)	32 (32.0%)	61 (45.9%)
Alcohol consumption, n (%)			
Non-drinker	50 (21.5%)	24 (24.0%)	26 (19.5%)
Drinker	125 (53.6%)	51 (51.0%)	74 (55.6%)
Ex-drinker	58 (24.9%)	25 (25.0%)	33 (24.9%)
FPG (mg/dL)	103.06 ± 21.34	94.25 ± 11.89	109.69 ± 24.32 ^a^
HbA1c (%)	5.98 ± 0.88	5.58 ± 0.511	6.29 ± 0.98 ^a^
TC (mg/dL)	199.14 ± 43.13	201.94 ± 40.81	194.03 ± 44.26
TG (mg/dL)	156.03 ± 108.16	126.80 ± 65.49	169.30 ± 79.50 ^a^
LDL-C (mg/dL)	131.30 ± 40.23	123.10 ± 41.39	136.89 ± 38.12 ^a^
HDL-C (mg/dL)	52.39 ± 13.16	57.04 ± 13.37	48.89 ± 11.90 ^a^
ALT (U/L)	27.24 ± 14.83	23.58 ± 13.32	30.07 ± 15.35 ^a^
AST (U/L)	25.35 ± 12.39	25.02 ± 14.98	25.60 ± 10.08
BUN (mg/dL)	13.57 ± 3.23	13.47 ± 3.50	13.64 ± 3.03
Creatinine (mg/dL)	1.01 ± 0.16	0.98 ± 0.13	1.04 ± 0.18

^a^ statistically significance difference (*p* < 0.05) from the non-obese group.

**Table 2:** DNA methylation of TNFα (GM ± SD) for each position and totals classified by metabolic components among non-obese and obese groups.

Study Group		DNA Methylation of TNFα				
		Position 1	Position 2	Position 3	Position 4	Total
Non-obese group (N = 100)		9.37 ± 2.21	8.14 ± 1.96	17.46 ± 3.89	23.16 ± 4.01	63.47 ± 8.99
Obese group (N = 133)		8.14 ± 2.01 ^a^	12.35 ± 3.12 ^a^	16.12 ± 4.08 ^a^	21.53 ± 3.97 ^a^	58.11 ± 10.22 ^a^
Metabolic syndrome-components						
Waist circumference						
Non-obese group	<90 cm (N = 62)	10.36 ± 2.24	13.20 ± 2.08	16.35 ± 3.58	21.32 ± 4.69	62.36 ± 8.74
	≥90 cm (N = 38)	9.33 ± 5.69	14.59 ± 3.14	17.02 ± 5.04	23.04 ± 5.07	61.99 ± 9.63
Obese group	<90 cm (N = 49)	8.99 ± 2.32	13.01 ± 4.02	17.25 ± 3.08	23.44 ± 3.99	62.48 ± 8.69
	≥90 cm (N = 84)	7.61 ± 2.14	11.05 ± 3.57 ^a^	15.37 ± 3.64 ^a^	21.09 ± 4.04 ^a^	57.96 ± 10.58 ^a^
HDL-cholesterol						
Non-obese group	<40 mg/dL (N = 83)	9.25 ± 3.95	13.33 ± 3.63	17.39 ± 4.16	22.91 ± 4.69	62.89 ± 12.62
	≥40 mg/dL (N = 10)	11.03 ± 2.85	15.21 ± 2.88	18.44 ± 4.57	26.42 ± 2.17	71.12 ± 7.35
Obese group	<40 mg/dL (N = 103)	8.01 ± 1.85	12.22 ± 2.71	16.03 ± 3.21	21.75 ± 4.26	58.02 ± 10.22
	≥40 mg/dL (N = 30)	8.58 ± 3.02	12.72 ± 3.49	16.40 ± 3.48	20.69 ± 4.67	58.41 ± 11.06
Triglyceride						
Non-obese group	<150 mg/dL (N = 73)	9.47 ± 4.12	13.35 ± 3.67	17.74 ± 4.27	23.11 ± 4.66	63.39 ± 12.95
	≥150 mg/dL (N = 26)	9.24 ± 3.28	13.91 ± 3.43	15.46 ± 3.76 ^a^	20.19 ± 4.69 ^a^	61.07 ± 11.06 ^a^
Obese group	<150 mg/dL (N = 71)	8.52 ± 2.47	12.97 ± 3.04	16.46 ± 3.52	22.45 ± 4.84	60.41 ± 11.45
	≥150 mg/dL (N = 62)	7.70 ± 1.67 ^a^	11.59 ± 2.54 ^a^	15.72 ± 2.92	20.43 ± 3.48 ^a^	55.46 ± 8.32 ^a^
High blood pressure (SBP ≥ 130 or DBP ≥ 85 mmHg						
Non-obese group	No (N = 45)	8.58 ± 3.66	13.41 ± 3.92	17.36 ± 3.88	22.96 ± 4.82	62.33 ± 11.98
	Yes (N = 55)	10.02 ± 4.00	13.50 ± 3.35	17.55 ± 4.43	23.3 ± 4.52	64.39 ± 12.90
Obese group	No (N = 30)	8.20 ± 2.18	12.33 ± 2.80	16.26 ± 3.22	21.63 ± 4.36	58.44 ± 10.01
	Yes (N = 103)	7.92 ± 2.15	12.32 ± 3.25	15.64 ± 3.41	19.46 ± 4.43	55.34 ± 11.67 ^a^
Fasting plasma glucose						
Non-obese group	<110 mg/dL (N = 85)	9.29 ± 3.89	13.39 ± 3.54	17.74 ± 4.25	23.03 ± 4.65	63.18 ± 12.39
	≥110 mg/dL (N = 15)	10.95 ± 4.14	14.72 ± 5.03	17.70 ± 2.64	25.49 ± 4.07	64.88 ± 14.40
Obese group	<110 mg/dL (N = 85)	8.09 ± 2.24	14.97 ± 2.91	16.17 ± 3.33	24.47 ± 4.45	61.36 ± 10.62
	≥110 mg/dL (N = 48)	8.21 ± 2.04	12.21 ± 2.89 ^a^	16.02 ± 3.15	21.79 ± 4.24 ^a^	58.25 ± 10.02 ^a^

^a^ significant different from non-obese group, *p* < 0.05.

**Table 3:** Univariate and multivariate analysis between DNA methylation of TNF-α and dietary factors among non-obese and obese groups.

Dietary Variables	Univariate Regression Analysis				Multivariate Regression Analysis			
	Non-Obese Group (N = 100)		Obese Group (N = 133)		Non-Obese Group (N = 100)		Obese Group (N = 133)	
	β	*p*-Value *	β	*p*-Value *	β	*p*-Value *	β	*p*-Value *
Frequency of red meat per week								
0–3 times	0	(<0.0001)	0	(0.005)	0	(0.057)	0	(0.024)
4–6 times	−3.98	<0.0001	−4.68	0.361	−1.21	0.071	−2.36	0.124
>6 times	−12.05	<0.0001	−11.14	<0.0001	−3.65	0.084	−10.25	<0.0001
Frequency of process meat per week								
0–3 times	0	(0.034)	0	(0.027)	0	(0.014)	0	(0.039)
4–6 times	−4.66	0.116	−2.29	0.417	−3.09	0.119	−1.47	0.097
>6 times	−7.61	<0.0001	−3.84	0.038	−5.23	0.001	−4.96	0.003
Frequency of fried meat per week								
0–3 times	0	(0.025)	0	(0.039)	0	(0.127)	0	(0.029)
4–6 times	−1.64	0.458	−2.81	0.660	−0.98	0.233	−1.29	0.068
>6 times	−12.79	0.004	−11.16	0.006	−1.07	0.057	−8.47	0.001
Frequency of fish per week								
0–3 times	0	(0.124)	0	(0.218)	0	(0.214)	0	(0.067)
4–6 times	−2.43	0.362	−0.85	0.737	1.96	0.198	1.14	0.314
>6 times	9.43	0.039	2.63	0.410	3.58	0.207	1.39	0.219
Frequency of egg per week								
0–3 servings	0	(0.207)	0	(0.011)	0	(0.127)	0	(0.041)
4–6 servings	1.18	0.865	7.66	0.002	0.95	0.141	3.04	0.053
>6 servings	4.68	0.478	6.78	0.012	2.14	0.097	5.99	0.041
Frequency of fruits per week								
0–3 servings	0	(0.008)	0	(0.019)	0	(0.039)	0	(0.017)
4–6 servings	4.34	0.062	8.57	0.001	2.36	0.612	6.32	0.001
>6 servings	7.17	0.014	9.32	<0.0001	4.96	0.027	7.18	<0.0001
Frequency of vegetables per week								
0–3 servings	0	(0.011)	0	(0.007)	0	(0.041)	0	(0.022)
4–6 servings	4.91	0.103	2.50	0.378	1.98	0.078	2.09	0.244
>6 servings	5.07	0.042	11.24	<0.0001	4.67	0.033	8.04	0.002

* The *p*-value in parentheses is the overall *p*-value for the diet variables, and the other *p*-values show comparisons to the reference category.

**Table 4:** Top ten downregulated and upregulated proteins with fold changes and *p*-values compared between the non-obese group and obese group (total proteins are described in Supplementary Table S1).

Protein ID	Protein Names	Gene Names	Gene Ontology (Biological Process)	log2 (FC)	*p*-Value
U5U6J5	Intercellular adhesion molecule 4	ICAM4	Cell adhesion [GO:0007155]	2.711	0.0022
B7Z339	Phosphatase and actin regulator			2.560	0.0010
Q8NEZ4	Histone-lysine N-methyltransferase 2C	KMT2C	Methylation [GO:0032259]; positive regulation of transcription by RNA polymerase II [GO:0045944];	2.538	0.0003
Q96RT7	Gamma-tubulin complex component 6 (GCP-6)	TUBGCP6	Cytoplasmic microtubule organization [GO:0031122]	2.531	0.0003
Q9BXL7	Caspase recruitment domain-containing protein 11	CARD11	Positive regulation of canonical NF-kappa b signal transduction; regulation of apoptotic process	2.523	0.0002
K7EML2	RNA binding motif protein 42	RBM42		2.522	0.0002
B1APH4	Putative zinc finger protein 487	ZNF487	Negative regulation of transcription by RNA polymerase II	2.500	0.0001
A0A1C7CYW9	Tubulin tyrosine ligase-like 8	TTLL8	Protein modification process	2.500	0.0002
F8WDP7	Cyclin-dependent kinase 15	CDK15		2.496	0.0002
Q96M60	Protein FAM227B	FAM227B		2.492	0.0002
Q9P2K1	Coiled-coil and C2 domain-containing protein 2A	CC2D2A	Axoneme assembly; smoothened signaling pathway	−2.6249	0.0009
A0A075B6T1	Autophagy and beclin 1 regulator 1	AMBRA1		−2.5975	0.0007
A0A590UJK2	Voltage-dependent P/Q-type calcium channel subunit alpha	CACNA1A	Regulation of monoatomic ion transmembrane transport	−2.4755	0.0001
Q7L0X2	Glutamate-rich protein 6	ERICH6		−2.4482	0.0001
Q8N523	Tuftelin-interacting protein 11	TFIP11	Biomineral tissue development	−2.4352	0.0000
A5YKK5	KIAA0232	KIAA0232		−2.4214	0.0001
G3V533	Spectrin repeat-containing nuclear envelope family member 3	SYNE3		−2.4202	0.0000
A0A2H4G345	MHC class II antigen	HLA-DQB1	Antigen processing and presentation immune response	−2.4183	0.0001
D6RGG8	Nicotinamide nucleotide adenylyltransferase 3	NMNAT3	Biosynthetic process	−2.4039	0.0000
A0A3B3IRX3	Mediator of RNA polymerase II transcription subunit 13	MED13L	Regulation of transcription by RNA polymerase II	−2.4033	0.0000

## Data Availability

The data presented in the current study are not publicly available owing to ethical restrictions. However, data are available from the corresponding author upon reasonable request.

## Supplementary Materials

The following supporting information can be downloaded at: https://www.mdpi.com/article/10.3390/nu16060877/s1, Table S1: Total of 276 significantly upregulated and downregulated proteins compared between the non-obese and obese groups.

## References

[1] Chew N.W.S., Ng C.H., Tan D.J.H., Kong G., Lin C., Chin Y.H., Lim W.H., Huang D.Q., Quek J., Fu C.E. (2023). The global burden of metabolic disease: Data from 2000 to 2019. Cell Metab..

[2] Samblas M., Milagro F.I., Martínez A. (2019). DNA methylation markers in obesity, metabolic syndrome, and weight loss. Epigenetics.

[3] Lomba A., Martínez J.A., García-Díaz D.F., Paternain L., Marti A., Campión J., Milagro F.I. (2010). Weight gain induced by an isocaloric pair-fed high fat diet: A nutriepigenetic study on FASN and NDUFB6 gene promoters. Mol. Genet. Metab..

[4] Ellulu M.S., Patimah I., Khaza’ai H., Rahmat A., Abed Y. (2017). Obesity and inflammation: The linking mechanism and the complications. Arch. Med. Sci..

[5] Tzanavari T., Giannogonas P., Karalis K.P. (2010). TNF-alpha and obesity. Curr. Dir. Autoimmun..

[6] Sethi J.K., Hotamisligil G.S. (2021). Metabolic Messengers: Tumour necrosis factor. Nat. Metab..

[7] Ramseyer V.D., Garvin J.L. (2013). Tumor necrosis factor-α: Regulation of renal function and blood pressure. Am. J. Physiol. Renal Physiol..

[8] Bollati V., Favero C., Albetti B., Tarantini L., Moroni A., Byun H.M., Motta V., Conti D.M., Tirelli A.S., Vigna L. (2014). Nutrients intake is associated with DNA methylation of candidate inflammatory genes in a population of obese subjects. Nutrients.

[9] Campión J., Milagro F.I., Goyenechea E., Martínez J.A. (2009). TNF-alpha promoter methylation as a predictive biomarker for weight-loss response. Obesity.

[10] Aleksandrova K., Egea Rodrigues C., Floegel A., Ahrens W. (2020). Omics biomarkers in obesity: Novel etiological insights and targets for precision prevention. Curr. Obes. Rep..

[11] Issaq H.J., Xiao Z., Veenstra T.D. (2007). Serum and plasma proteomics. Chem. Rev..

[12] Cominetti O., Galindo A.N., Corthésy J., Valsesia A., Irincheeva I., Kussmann M., Saris W.H.M., Astrup A., McPherson R., Harper M.-E. (2018). Obesity shows preserved plasma proteome in large independent clinical cohorts. Sci. Rep..

[13] Zaghlool S.B., Kühnel B., Elhadad M.A., Kader S., Halama A., Thareja G., Engelke R., Sarwath H., Al-Dous E.K., Mohamoud Y.A. (2020). Epigenetics meets proteomics in an epigenome-wide association study with circulating blood plasma protein traits. Nat. Commun..

[14] Lau F.C., Bagchi M., Sen C., Roy S., Bagchi D. (2008). Nutrigenomic analysis of diet-gene interactions on functional supplements for weight management. Curr. Genom..

[15] Huang P.L. (2009). A comprehensive definition for metabolic syndrome. Dis. Model. Mech..

[16] Ng C. (2019). Stratification of BMI categories among older adults within and across countries. Public Health Nutr..

[17] Kerdsaeng N., Roytrakul S., Chanprasertyothin S., Charernwat P., Chansirikarnjana S., Sritara P., Sirivarasai J. (2021). Serum glycoproteomics and identification of potential mechanisms underlying Alzheimer’s disease. Behav. Neurol..

[18] Tansakul N., Rattanasrisomporn J., Roytrakul S. (2019). Proteomics analysis of serum protein patterns in duck during aflatoxin B1 exposure. Vet. World.

[19] Moore L.D., Le T., Fan G. (2013). DNA methylation and its basic function. Neuropsychopharmacology.

[20] Hermsdorff H.H., Mansego M.L., Campión J., Milagro F.I., Zulet M.A., Martínez J.A. (2013). TNF-alpha promoter methylation in peripheral white blood cells: Relationship with circulating TNFα, truncal fat and n-6 PUFA intake in young women. Cytokine.

[21] Lim U., Song M.A. (2012). Dietary and lifestyle factors of DNA methylation. Methods Mol. Biol..

[22] Cӑtoi A.F., Pârvu A.E., Andreicuț A.D., Mironiuc A., Crӑciun A., Cӑtoi C., Pop I.D. (2018). Metabolically healthy versus unhealthy morbidly obese: Chronic inflammation, nitro-oxidative stress, and insulin resistance. Nutrients.

[23] Mazor R., Itzhaki O., Sela S., Yagil Y., Cohen-Mazor M., Yagil C., Kristal B. (2010). Tumor necrosis factor-alpha: A possible priming agent for the polymorphonuclear leukocyte-reduced nicotinamide-adenine dinucleotide phosphate oxidase in hypertension. Hypertension.

[24] Akash M.S.H., Rehman K., Liaqat A. (2018). Tumor necrosis factor-alpha: Role in development of insulin resistance and pathogenesis of type 2 diabetes mellitus. J. Cell. Biochem..

[25] Du X., Liu M., Tai W., Yu H., Hao X., Loor J.J., Jiang Q., Fang Z., Gao X., Fan M. (2022). Tumor necrosis factor-α promotes lipolysis and reduces insulin sensitivity by activating nuclear factor kappa B and c-Jun N-terminal kinase in primary bovine adipocytes. J. Dairy Sci..

[26] Schwedhelm C., Pischon T., Rohrmann S., Himmerich H., Linseisen J., Nimptsch K. (2017). Plasma inflammation markers of the tumor necrosis factor pathway but not C-reactive protein are associated with processed meat and unprocessed red meat consumption in Bavarian adults. J. Nutr..

[27] Sajadi Hezaveh Z., Sikaroudi M.K., Vafa M., Clayton Z.S., Soltani S. (2019). Effect of egg consumption on inflammatory markers: A systematic review and meta-analysis of randomized controlled clinical trials. J. Sci. Food Agric..

[28] Ballesteros M.N., Valenzuela F., Robles A.E., Artalejo E., Aguilar D., Andersen C.J., Valdez H., Fernandez M.L. (2015). One egg per day improves inflammation when compared to an oatmeal-based breakfast without increasing other cardiometabolic risk factors in diabetic patients. Nutrients.

[29] Gariballa S., Al-Bluwi G.S.M., Yasin J. (2023). Increased fruit and vegetable consumption mitigates oxidative damage and associated inflammatory response in obese subjects independent of body weight change. Nutrients.

[30] Hermsdorff H.H., Zulet M.A., Puchau B., Martínez J.A. (2010). Fruit and vegetable consumption and proinflammatory gene expression from peripheral blood mononuclear cells in young adults: A translational study. Nutr. Metab..

[31] Quetglas-Llabrés M.M., Monserrat-Mesquida M., Bouzas C., Mateos D., Ugarriza L., Gómez C., Tur J.A., Sureda A. (2023). Oxidative stress and inflammatory biomarkers are related to high intake of ultra-processed food in old adults with metabolic syndrome. Antioxidants.

[32] Maugeri A., Barchitta M. (2020). How dietary factors affect DNA methylation: Lesson from epidemiological studies. Medicina.

[33] Clemente-Suárez V.J., Martín-Rodríguez A., Redondo-Flórez L., López-Mora C., Yáñez-Sepúlveda R., Tornero-Aguilera J.F. (2023). New insights and potential therapeutic interventions in metabolic diseases. Int. J. Mol. Sci..

[34] Morris R., Kershaw N.J., Babon J.J. (2018). The molecular details of cytokine signaling via the JAK/STAT pathway. Protein Sci..

[35] Savova M.S., Mihaylova L.V., Tews D., Wabitsch M., Georgiev M.I. (2023). Targeting PI3K/AKT signaling pathway in obesity. Biomed. Pharmacother..

[36] Ihanus E., Uotila L.M., Toivanen A., Varis M., Gahmberg C.G. (2007). Red-cell ICAM-4 is a ligand for the monocyte/macrophage integrin CD11c/CD18: Characterization of the binding sites on ICAM-4. Blood.

[37] Campbell K.L., Foster-Schubert K.E., Makar K.W., Kratz M., Hagman D., Schur E.A., Habermann N., Horton M., Abbenhardt C., Kuan L.Y. (2013). Gene expression changes in adipose tissue with diet- and/or exercise-induced weight loss. Cancer Prev. Res..

[38] Pomerantz J.L., Denny E.M., Baltimore D. (2002). CARD11 mediates factor-specific activation of NF-kappaB by the T cell receptor complex. EMBO J..

[39] Hildebrandt X., Ibrahim M., Peltzer N. (2023). Cell death and inflammation during obesity: “Know my methods, WAT(son)”. Cell Death Differ..

[40] Pan C., Lei Z., Wang S., Wang X., Wei D., Cai X., Luoreng Z., Wang l., Ma Y. (2021). Genome-wide identification of cyclin-dependent kinase (CDK) genes affecting adipocyte differentiation in cattle. BMC Genom..

[41] Cianfanelli V., Nazio F., Cecconi F. (2015). Connecting autophagy: AMBRA1 and its network of regulation. Mol. Cell. Oncol..

[42] Li X., Lyu Y., Li J., Wang X. (2022). AMBRA1 and its role as a target for anticancer therapy. Front. Oncol..

[43] Hu Z., Liu F., Li M., He J., Huang J., Rao D.C., Hixson J.E., Gu C., Kelly T.N., Chen S. (2016). Associations of variants in the CACNA1A and CACNA1C genes with longitudinal blood pressure changes and hypertension incidence: The GenSalt Study. Am. J. Hypertens..

[44] Johnson A.D., Newton-Cheh C., Chasman D.I., Ehret G.B., Johnson T., Rose L., Rice K., Verwoert G., Launer L.J.C., Gudnason V. (2011). Cohorts for heart and aging research in genomic epidemiology consortium; Global BPgen Consortium; Women’s Genome Health Study. Association of hypertension drug target genes with blood pressure and hypertension in 86,588 individuals. Hypertension.

[45] Deng T., Lyon C.J., Minze J., Lin J., Zou J., Liu J.Z., Ren Y., Yin Z., Hamilton D.J., Reardon P.R. (2013). Class II major histocompatibility complex plays an essential role in obesity-induced adipose inflammation. Cell Metab..

